# Neonatal scrotal wall necrotizing fasciitis (Fournier gangrene): a case report

**DOI:** 10.1186/1752-1947-5-576

**Published:** 2011-12-12

**Authors:** Oskar Zgraj, Sri Paran, Maureen O'Sullivan, Feargal Quinn

**Affiliations:** 1 Department of Paediatric Surgery, Our Lady's Children's Hospital, Crumlin, Dublin 12, Ireland; 2 Department of Pathology, Our Lady's Children's Hospital, Crumlin, Dublin 12, Ireland

## Abstract

**Introduction:**

Necrotizing fasciitis in neonates is rare and is associated with almost 50% mortality. Although more than 80 cases of neonates (under one month of age) with necrotizing fasciitis have been reported in the literature, only six of them are identified as originating in the scrotum.

**Case presentation:**

We report the case of a four-week-old, full-term, otherwise-healthy Caucasian baby boy who presented with an ulcerating lesion of his scrotal wall. His scrotum was explored because of a provisional diagnosis of missed torsion of the testis. He was found to have necrotizing fasciitis of the scrotum. We were able to preserve the testis and excise the necrotic tissue, and with intravenous antibiotics there was a successful outcome.

**Conclusions:**

Fournier gangrene is rarely considered as part of the differential diagnosis in the clinical management of the acute scrotum. However, all doctors who care for small babies must be aware of this serious condition and, if it is suspected, should not hesitate in referring the babies to a specialist pediatric surgical center immediately.

## Introduction

Testicular torsion is a surgical emergency and is commonly seen in children between five and 15 years of age. Neonatal torsion of the testis is a well-recognized condition in which an extravaginal torsion of the testis is known to occur before birth and invariably is associated with a necrotic testis [1]. A firm and often painless testicular mass is detected at birth, with or without skin discoloration and edema.

Necrotizing fasciitis in neonates is extremely rare and is associated with almost 50% mortality [2]. Although more than 80 cases of neonates (under one month of age) with necrotizing fasciitis have been reported in the literature [2], only six of them are identified as originating in the scrotum [3-7]. There are also reports of peritesticular abscess mimicking or resulting from neonatal torsion [8,9]. Fournier gangrene is rarely considered as part of the differential diagnosis in the clinical management of the acute scrotum.

## Case presentation

A 10-day-old, full-term Caucasian baby boy presented with viral gastroenteritis to his local hospital. During admission, pediatric surgical services were consulted for left scrotal swelling and a diagnosis of a left hydrocele was made and patient was discharged. Seventeen days later (at four weeks of age), he presented with a 24-hour history of left scrotal swelling, erythema, pyrexia, and a pus-like discharge from an ulcerated area in his left hemi-scrotum. The results of an abdominal examination were normal, and a scrotal examination revealed a left scrotal abscess with a hard swollen left testis. No hernia was identified. A provisional diagnosis of missed neonatal testicular torsion was made and the baby was prepared for immediate surgical exploration. Intravenous antibiotics (amoxicillin with clavulanic acid and flucloxacillin) and fluids were commenced in light of the local scrotal infection.

A midline raphe scrotal incision was used. A small amount of pus was evacuated, and a necrotic scrotal wall with an appearance similar to that of Fournier gangrene was excised back to the healthy tissue margin. The excised tissue was sent for histology and microbiology. The tunica vaginalis was thickened, and the testis was found to be edematous with normal blood supply. There was no evidence of testicular torsion. The wound was thoroughly washed with antiseptic solution. The dartos muscle and skin were closed primarily by means of absorbable sutures. The pyrexia was noted to settle soon after surgery. Edema, erythema, and pain settled over the following 48 hours.

**Figure 1:**
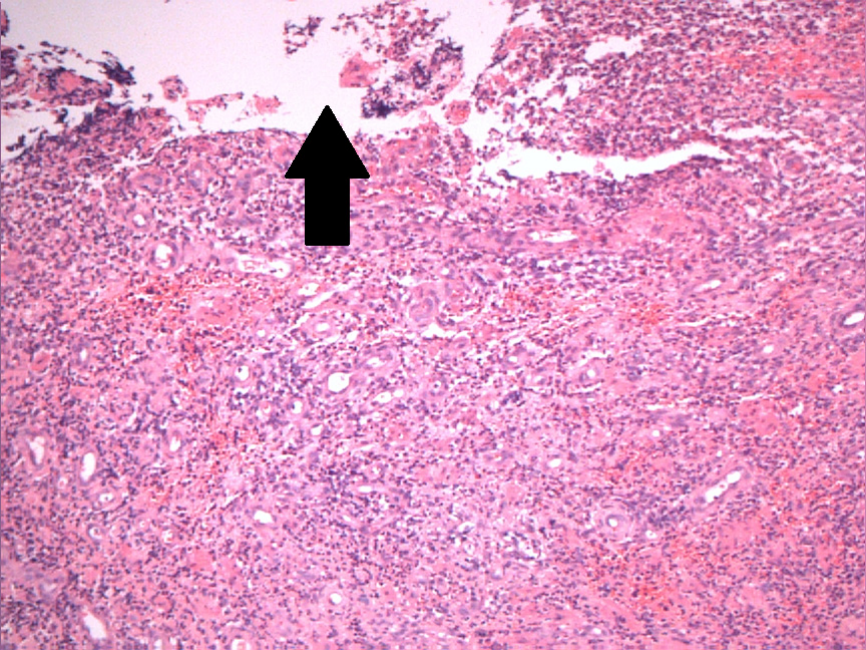
**Histological findings (optical microscopy eosin-hematoxilin) of a four-week-old boy with necrotizing fasciitis of the scrotal wall**. Ulcerated granulation tissue is shown. The arrow points to absent epidermis, indicating ulceration. Sections of hyper- and parakeratotic scrotal skin gave way to extensive ulceration. The dermis was replaced by remarkably inflamed granulation tissue and essentially comprised only capillaries and pus in many areas. The connective tissue was focally preserved adjacent to these necrotic foci. There was extensive ulceration of the surface also. No granulomas were identified. The appearance was that of necrotizing acute inflammation of the scrotal skin (Fournier gangrene).

Wound cultures revealed *Enterococcus *sp. (ampicillin-sensitive) and *Staphylococcus aureus *(methicillin-sensitive). The antibiotic regime mentioned above was continued for a total of 14 days (five days intravenously and the remainder orally). The baby was discharged home on the fifth post-operative day with oral antibiotics. Histology confirmed the intra-operative diagnosis (Figure 1). The baby has remained well, and wound healing was normal at follow-up in an out-patient clinic at two weeks, two months, and six months. The results of a scrotal examination each time were normal.

## Discussion

Fournier gangrene is more common in adults - peak incidence occurs between 20 and 50 years of age - and is extremely rare in children. Only around 80 cases of necrotizing fasciitis have been reported in the literature [2-4]. Fournier gangrene can affect any part of the body in children, but the trunk and perineum are commonly affected in newborns. Like adults, preterm and low-birth weight babies with impaired immune status and those with poor local hygiene appear to have increased risk of this rare disease. An initial injury to the skin was documented in the majority of children but was not noted or reported in up to 40% of cases in some studies [10].

When the skin barrier is breached, the organisms appear to spread into the subcutaneous tissue and produce fascial necrosis with an obliterative endarteritis leading to further necrosis of tissue. The disease process, like that of our patient, is usually outside the tunica and hence the blood supply via the testicular artery is preserved. Although a left-sided hydrocele was diagnosed 17 days prior to this admission, no obvious hydrocele was noted at the time of surgery. Whether the initial swelling was due to a subacute phase of the disease process or truly was an isolated hydrocele that resolved spontaneously over this short period of time is not clear.

Neonatal testicular torsion is a well-recognized condition. Patients present with unilateral, often painless scrotal swelling and erythema of overlying skin. The management of this condition is controversial. Some surgeons recommend immediate exploration whereas others prefer conservative management [8]. Paratesticular abscess (or scrotal abscess) has been reported to mimic neonatal torsion. We elected to proceed to immediate surgical exploration in this baby because the possibility of a missed torsion complicated by a scrotal abscess could not be safely ruled out. This immediate surgical approach allowed us to address the unexpected Fournier gangrene early with minimal soft tissue excision and primary wound closure. No radiological investigations were performed, as the presence of an abscess and systemic signs of sepsis necessitated immediate surgical exploration.

Although the initial literature advocates early aggressive surgical debridement of Fournier gangrene wounds, a recent report shows a successful outcome with a more conservative and selective surgical debridement [4]. When small babies with acute onset of scrotal swelling are assessed, it is important to exclude testicular pathology. If an experienced pediatric surgeon is not readily available, an ultrasound scan may be useful to exclude common pathologies such as an obstructed hernia, tense hydrocele, or hydrocele of the cord. When the testis is clearly palpable with no obvious pathology and scrotal wall edema or erythema is the main finding, Fournier gangrene should be considered in the differential diagnosis. We recommend an early surgical approach, first to exclude torsion of the testis and then to debride the involved region. However, the need for aggressive wide debridement appears to be unnecessary in localized disease as long as adequate antibiotic coverage is provided. We recommend more than one antibiotic coverage, including one specifically targeted toward *S. aureus*. Both *Enterococcus *and *S. aureus *have been implicated as the causative organisms of Fournier gangrene in previous reports [2,11].

## Conclusions

Fournier gangrene of the scrotum is extremely rare in newborns but should be considered as part of the differential diagnosis in the clinical management of the acute scrotum. If it is considered a possibility, then immediate referral to a dedicated pediatric surgical unit is indicated.

## Consent

Written informed consent was obtained from the patient's parents for publication of this case report and accompanying images. A copy of the written consent is available for review by the Editor-in-Chief of this journal.

## Competing interests

The authors declare that they have no competing interests.

## Authors' contributions

OZ gathered patient data, performed a literature search, calculated data from previous articles, and wrote the core of the manuscript. SP analyzed the patient's presentation in detail and made a major contribution to the manuscript, especially the Discussion section. MO'S performed a histological examination of specimens, photographed them, prepared the figure and legend, contributed in interpreting histological findings in view of the clinical presentation, and made corrections to the manuscript with special consideration to international histological nomenclature.

FQ was the consultant in charge of this case, made the intra-operative diagnosis, directed the management, and finalized the manuscript, shaping the final conclusions. All authors read and approved the final manuscript.
